# Adjusting urinary chemical biomarkers for hydration status during pregnancy

**DOI:** 10.1038/s41370-018-0043-z

**Published:** 2018-06-08

**Authors:** Susan MacPherson, Tye E. Arbuckle, Mandy Fisher

**Affiliations:** Population Studies Division, Environmental Health Science and Research Bureau, Healthy Environments and Consumer Safety Branch, Health Canada, Ottawa, ON Canada

**Keywords:** pregnancy, urinary dilution, creatinine, specific gravity

## Abstract

One way of assessing a population’s exposure to environmental chemicals is by measuring urinary biomarker concentrations, which can vary depending on the hydration status of the individual. The physiological changes that occur during pregnancy can impact the hydration adjustment approaches, such as calculating the individual’s urinary flow rate (UFR), or adjusting concentrations using specific gravity (SG) or creatinine. A total of 1260 serial spot urine samples were collected from 80 women, averaging 32.4 years of age, throughout and shortly after pregnancy. The relationship between each approach was examined and time of day and across pregnancy differences were tested using linear mixed models. The correlation between the calculated excretion rate and each of the adjustment techniques was examined on a selection of seven phthalate metabolites. Based on the linear mixed model results, we found that UFR and creatinine excretion rates differed systematically across the population, with respect to body mass index (BMI) and time. SG differed with respect to BMI, but there were no systematic time trends. SG had the highest within-person reproducibility, according to the intraclass correlation coefficient (ICC). The excretion rate of each of the phthalates was most strongly correlated with the SG-standardized concentration. This analysis showed that SG showed a slightly better within-person reproducibility and the least amount of systematic variation when compared to creatinine adjustment. Therefore, SG correction appears to be a favorable approach for correcting for the hydration status of the pregnant women from this cohort.

## Introduction

Exposure to environmental chemicals is often assessed through the direct measurement of urinary biomarker concentrations. The hydration status and physiological differences among study participants must be considered to estimate the exposures from these measurements. One reliable option to account for the differences in hydration status is to collect all urine samples throughout a 24 h period together and measure the biomarker concentration in the composite samples to derive average biomarker excretion per day. Although this is regarded as the most reliable method and is less influenced by physiological factors, 24 h collections could lead to underestimation of the results when studying short-lived chemicals [1]. The concentration (but not mass) of an analyte measured shortly after exposure can be diluted with samples collected after the chemical has been mostly eliminated. Although collections over shorter periods may be more influenced by diurnal variations in excretion [2], spot urine samples are easier to collect, more cost effective and minimize subject compliance concerns [3].

To interpret biomonitoring results in spot urine samples, normalization techniques have been applied because the variation in concentrations can only, in part, be assessed from the differences in exposure levels. Other important sources of variation to consider are the timing between exposure and when the urine sample is collected, pharmacokinetics, the half-life of the chemical and the substantial variation in hydration status. To account for the hydration status directly, the average excretion rate of a biomarker can be calculated by multiplying its concentration in a spot sample by the urinary flow rate which, in the context of biomonitoring, is calculated by dividing the total volume of the urine sample by the total time since the previous void. When the urinary flow rate cannot be calculated directly, variations within and between individuals, as well as within and across days, can cloud the interpretation of biomonitoring results [4, 5]. As a result, a number of approaches to adjust spot urinary concentrations for hydration status have been used as surrogates, principal among them creatinine correction and specific gravity adjustment. For a measure to be useful as a correction factor, it should not systematically vary across demographic groups of interest, such as age or sex, or across time [6]. It is important to understand how these factors relate to a population, particularly a unique population such as pregnant women, to ensure differences among individuals, across time or demographic variables are due to the levels of exposure and not exaggerated by the correction factors themselves.

During pregnancy a number of physiological changes occur that may affect the interpretation of urinary biomarkers. Glomerular filtration rate (GFR), which describes the flow rate of filtered fluid through the kidney, increases by 50%, tubular function and handling of water are altered and the kidneys grow larger [7]. In the first trimester of pregnancy, 24 h urine output and creatinine excretion increases from about week 4 onwards. During the third trimester, there appears to be a downward trend of about 16%. This may indicate an alteration in renal handling of creatinine or a genuine reduction in the GFR [8, 9].

Weaver et al. [2] recommend comparing different urine concentration adjustment methods from the same dataset, and when these adjustment methods are not highly correlated, trying to identify the factors involved. The design of the Plastics and Personal-care Products use in Pregnancy (P4) study permitted examination of these factors in a cohort of pregnant women.

The data provided by this study enabled us to evaluate and compare the normalization techniques specific to the pregnant population. There were two main objectives of the study. The first was to summarize and assess relationships between various measures relating to hydration status in our pregnant population, including specific gravity, creatinine, urinary flow rate and the creatinine excretion rate. The second was to find the most favorable approach to correct for the hydration status of the pregnant women. We used the following three criteria to select the best adjustment method in our study. (1) To be a useful correction factor, a measure should be subject to only minimal intra-individual variations, so we calculated the intraclass correlation coefficients (ICCs) using a random intercept mixed model to find which measure had the highest within-person reproducibility. (2) A correction factor should remain constant day-to-day and be little influenced by demographic characteristics, so we implement a mixed-effects model for each measure, to test for time of day and across pregnancy differences in urinary levels, as well as to test for associations between each of the metrics and several demographic variables. (3) To examine which surrogate adjustment approach best correlates to the calculated excretion rate, we selected seven phthalate metabolites and assessed the correlation between the specific gravity and creatinine-standardized concentrations and the calculated excretion rate. The phthalate metabolites were monoethyl phthalate (MEP), mono-*n*-butyl phthalate (MBP), monobenzyl phthalate (MBzP), monoethylhexyl phthalate (MEHP), mono-2-ethyl-5-hydroxyhexyl phthalate (MEHHP), mono-2-ethyl-5-oxohexyl (MEOHP) and mono-3-carboxypropyl phthalate (MCPP).

## Methods

### Study participants

The P4 study recruited women from obstetrical clinics in Ottawa, Canada, between November 2009 and December 2010. The study was approved by human studies research ethics committees at Health Canada and all participating hospitals. Eligible women had to be over 18 years of age, able to communicate in English or French and planning on delivering locally. Women who had fetal abnormalities or major malformations in the current pregnancy, or had medical complications (e.g., renal disease with altered renal function, active or chronic hepatitis, thyroid disorder, hypertension, diabetes and epilepsy), threat of spontaneous abortion, illicit drug use or were already participating in two or more research studies were excluded from the study. Sample size calculations indicated that we required a minimum of 15 women to obtain 80% power to estimate the within-subject variance with an error of less than 0.06. There were 80 women who signed consent forms and were followed prospectively through pregnancy and up to 2–3 months postnatally. Participants completed a short questionnaire at recruitment and at each contact throughout the study. The questionnaire collected information on occupation, socio-economic status, obstetrical history, smoking and details relating to the current pregnancy. Further information on the P4 study has been described elsewhere [10, 11].

### Urine collection

The women were asked to collect all urine voids over a 24 h period on a weekday (T1A) and optionally on a weekend day (T1B) during early pregnancy (<20 weeks), as well as a spot urine void during the 2nd (T2) and 3rd (T3) trimesters and again 2–3 months postpartum (T5). Women were asked to collect and record the dates and times of all urine voids over the 24 h periods (T1A, T1B). For the single spot urine voids (T2, T3 and T5), the time of the void and the time since last void were noted. Urine was collected in prescreened urine cups (polypropylene) and kept cool (4 °C) to avoid degradation of the chemical until aliquoted and stored at −80 °C.

### Measurements and calculations

To assess urine dilution, the urinary flow rate (UFR), which corresponds to the volume of urine excreted on average per unit of time (ml/h), as well as the bodyweight-adjusted urinary flow rate (UFR_BW), were calculated for each sample as follows:

1 $${{\mathrm{{UFR}}}}({{\mathrm{{ml}}}}{\mathrm{/}}{{\mathrm{{h}}}}) = \frac{{{{\mathrm{{Void}}}}\,{{\mathrm{{volume}}}}\,({{\mathrm{{ml}}}})}}{{{{\mathrm{{Time}}}}\,{{\mathrm{{since}}}}\,{{\mathrm{{last}}}}\,{{\mathrm{{void}}}}\,({{\mathrm{{h}}}})}}.$$

2 $${{\mathrm{{UFR}}}}\_{{\mathrm{{BW}}}}\,({{\mathrm{{ml}}}}{\mathrm{/}}{{\mathrm{{h}}}} - {{\mathrm{{kg}}}}) = \frac{{{{\mathrm{{Void}}}}\,{{\mathrm{{volume}}}}\,({{\mathrm{{ml}}}})}}{{{{\mathrm{{Time}}}}\,{{\mathrm{{since}}}}\,{{\mathrm{{last}}}}\,{{\mathrm{{void}}}}\,\left( {{{\mathrm{{h}}}}} \right) \ast {{\mathrm{{BW}}}}\,({{\mathrm{{kg}}}})}}.$$

Creatinine (CRE), a byproduct of muscle activity, is cleared from the bloodstream by the kidneys and excreted in urine. The creatinine concentration was determined based on the Jaffe method. The creatinine excretion rate (ER_CRE) and the bodyweight-adjusted creatinine excretion rate (ER_CRE_BW) were calculated using the following formulas:

3 $${{\mathrm{{ER}}}}\_{{\mathrm{{CRE}}}}\,({{\mathrm{{mg}}}}{\mathrm{/}}{{\mathrm{{h}}}}) = {{\mathrm{{CRE}}}}\,({{\mathrm{{mg}}}}{\mathrm{/}}{{\mathrm{{dl}}}}) \ast {{\mathrm{{UFR}}}}\,({{\mathrm{{dl}}}}{\mathrm{/}}{{\mathrm{{h}}}}).$$

4 $${{\mathrm{{ER}}}}\_{{\mathrm{{CRE}}}}\_{{\mathrm{{BW}}}}\,({{\mathrm{{mg}}}}{\mathrm{/}}{{\mathrm{{h}}}} - {{\mathrm{{kg}}}}) = \frac{{{{\mathrm{{ER}}}}\_{{\mathrm{{CRE}}}}\,({{\mathrm{{mg}}}}{\mathrm{/}}{{\mathrm{{h}}}})}}{{{{\mathrm{{BW}}}}\,({{\mathrm{{kg}}}})}}.$$

Studies measuring phthalate metabolite levels in urine demonstrate ongoing exposures to phthalates in the general population, as well as subpopulations, such as pregnant women [12]. To demonstrate which surrogate adjustment approach best correlates with the excretion rate of a biomarker, we chose a selection of phthalates for which we had data for all pregnancy time points. Assumed to have a short elimination half-life [13], the timing between phthalate exposure and urine sampling can greatly influence the measured concentration. The analytical method for phthalate analysis has been previously described [11]. The “true” excretion rate of each phthalate (ER_CHEM) is calculated directly by multiplying the concentration by the urine flow rate:

5 $${{\mathrm{{ER}}}}\_{{\mathrm{{CHEM}}}}\, ({{\mathrm{{mg}}}}{{/}}{{\mathrm{{h}}}}) = {{\mathrm{{CHEM}}}}\,({{\mathrm{{mg}}}}{\mathrm{/}}{{\mathrm{{dl}}}}) \ast {{\mathrm{{UFR}}}}\,({{\mathrm{{dl}}}}{\mathrm{/}}{{\mathrm{{h}}}}).$$

The creatinine-standardized concentrations were calculated as the ratio of the chemical and the creatinine concentrations and expressed in μg/g creatinine:

6 $${{\mathrm{{CHEM}}}}_{{{\mathrm{{CRE}}}} - {{\mathrm{{Adj}}}}}\,({{\mathrm{{\mu}}} {{\mathrm{{g}}}}}{\mathrm{/}}{{\mathrm{{g}}}}\,{{\mathrm{{CRE}}}}) = \frac{{{{\mathrm{{CHEM}}}}\,({{\mathrm{{\mu}}} {\mathrm{g}}}{\mathrm{/}}{l})}}{{{{\mathrm{{CRE}}}}({{\mathrm{{g}}}}\,{{\mathrm{{CRE}}}}{\mathrm{/}}{l})}}.$$

Specific gravity (SG) is the ratio of the density of a urine specimen to the density of water and was measured using a refractometer with automatic temperature compensation, on urine that had undergone a freeze–thaw cycle. Specific gravity-standardized analyte concentrations were calculated using the following formula: [14]

7 $${{\mathrm{{CHEM}}}}_{{{\mathrm{{SG}}}}\_{{\mathrm{{Adj}}}}}\,({{\mathrm{{\mu}} \mathrm {g}}}{\mathrm{/}}{{\mathrm{{l}}}}) = {{\mathrm{{CHEM}}}}_{{\mathrm{{i}}}}\frac{{({{\mathrm{{SG}}}}_{{\mathrm{{m}}}}-1)}}{{({{\mathrm{{SG}}}}_{{\mathrm{{i}}}}-1)}},$$

where ${\mathrm{{CHEM}}}_{\mathrm{{SG}}}\_{\mathrm{{Adj}}}$ is the specific gravity-standardized analyte concentration (µg per l), ${\mathrm{{CHEM}}}_{\mathrm{{i}}}$ is the observed analyte concentration, SG_i_ is the specific gravity of the urine sample and SG_m_ is the median specific gravity for the cohort.

### Statistical analysis

Summary statistics for each of the time points were calculated for urinary flow rates, creatinine excretion rates, specific gravity and creatinine concentrations. The bodyweight-adjusted results were also calculated for each time point, with the exception of T5 (2–3 months postpartum), in which the mother’s bodyweight was not measured. To test for associations between each of the metrics and a selection of demographic variables, we implemented mixed-effects models with random subject effects to account for potential correlation of repeated measurements within an individual. The covariates, tested one at a time, were selected a priori and included maternal age (<30, 30–35, >35), body mass index) (BMI) at the time of urine collection (underweight/normal, overweight/obese), parity (0, 1, 2+), household income (<$100K, ≥$100K), education level (<college, college diploma, bachelors, masters/PhD) smoking status (never, ever) and whether the participant was Canadian born (yes, no).

We also tested for time of day and across pregnancy differences in urinary levels within individuals using a mixed-effects multiple regression approach to adjust for any significant covariates. Given that all urinary levels, except for specific gravity, were not normally distributed, values were natural log transformed prior to testing. Mixed model estimates were produced using restricted maximum likelihood (REML) estimation with an unstructured covariance structure, and *p* values were constructed using the Kenward Roger degrees of freedom method. Heat maps and scatterplots were depicted to help visualize the diurnal pattern of hydration during pregnancy. The nature of the relationship between specific gravity and creatinine was explored graphically using a scatterplot for each pregnancy period.

ICCs were calculated using a random intercept mixed model to estimate the between- and within-subject variability within a day and throughout pregnancy. The ICC measures the ratio of between-subject variance to total variance ranging from 0, meaning no within-person reproducibility, to 1, meaning perfect reproducibility. Any value above 0.75 is defined as high, 0.40–0.75 as moderate and below 0.40 is defined as having poor reproducibility [15]. The ICCs were calculated for samples collected throughout a weekday, a weekend day, across pregnancy and across all study time points. The 95% confidence intervals for the ICCs were based on the methods of Hankinson et al. [16].

To further compare each adjustment approach, we applied the correction methods to seven different phthalates and calculated the geometric means and the ICCs to see how the reproducibility changes after concentrations have been standardized for hydration. Additionally, Pearson's correlation coefficients between the natural log transformed excretion rates, unadjusted, specific gravity-standardized and creatinine-standardized phthalate concentrations were assessed to evaluate which adjustment surrogate best correlates with the true excretion rate. This allowed us to explore the applicability of specific gravity and creatinine as normalization methods when adjusting phthalate metabolites in the pregnant population. Data analysis was performed using SAS Enterprise Guide (version 5.1). Due to privacy issues, supporting data cannot be made openly available. Further information about the data and the computer code that supports the findings of this study is available from the corresponding author upon reasonable request.

## Results

**Fig. 1:**
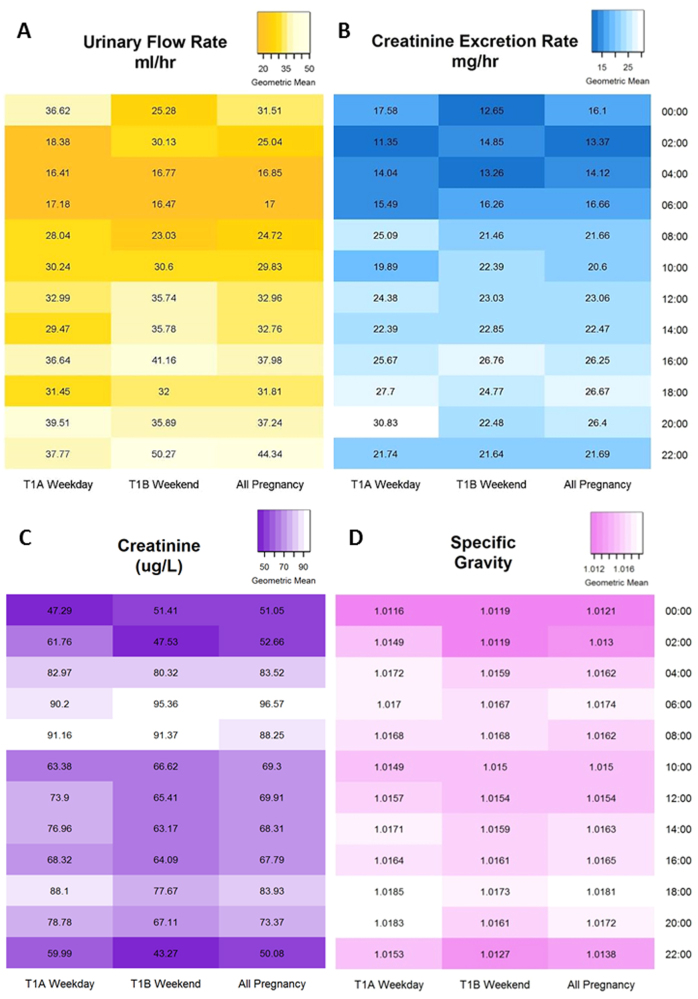
**Geometric means by time of day**. Heat maps depicting the geometric means by time of day for T1A: <20 weeks gestation week day, T1B: <20 weeks gestation weekend day and throughout pregnancy for (a) urinary flow rate (ml/hr), (b) excretion rate of creatinine (mg/hr), (c) creatinine concentration (µg/L) and (d) specific gravity

A total of 1260 urine samples were collected throughout and shortly after pregnancy, with an average of 3 h between urine voids. Collected voids were aliquoted and frozen within 10 min to 60 h after collection, with a median time of 19.8 h. The total volume of each sample ranged from 10 to 120 ml, with a median volume of 70 ml and an average (standard deviation) of 73.8 (29.3) ml. Descriptive statistics, including arithmetic means (AM), geometric means (GM) with 95% confidence limits (CL) and select percentiles for urinary flow rates, creatinine excretion rates bodyweight-adjusted rates, specific gravity and creatinine concentrations, by time point, are shown in Table 1. All creatinine concentrations exceeded the limit of detection. Urinary flow and creatinine excretion rates were highest during the first trimester of pregnancy, inverse to creatinine concentration, which was lowest during the first trimester. To evaluate the diurnal pattern of hydration during pregnancy, the heat map depicted in Fig. 1a shows that urinary flow rates varied greatly by time of day. The lowest urinary flow rates, which represent the most concentrated urine, occurred during the nighttime hours, between midnight and 8:00 a.m. The excretion rate of creatinine by time of day, shown in Fig. 1b, was calculated using urinary flow rate and therefore followed a similar pattern, with the lower rates occurring overnight. The heat map in Fig. 1c shows that creatinine concentration varies inversely with urinary flow rate as the highest concentrations occur during the day. Figure 1d shows that specific gravity does not seem to vary as much as the others, by time of day.

**Table 1:** Descriptive statistics of urinary flow rate, creatinine excretion rate, specific gravity and creatinine concentrations, by sampling time point

	# Obs	Min	5th percentile	Median	95th percentile	Max	AM	GM	GM 95% lower CL	GM 95% upper CL
UFR (ml/h)										
T1A	475	2.878	9.231	28.966	100.000	720.000	39.554	29.486	27.608	31.491
T1B	498	0.444	7.973	31.716	125.000	480.000	44.116	31.200	29.010	33.556
T2	54	3.214	5.405	20.435	64.615	140.000	27.848	20.537	16.475	25.600
T3	57	1.290	5.769	22.222	100.000	165.000	29.048	20.938	16.762	26.154
T5	48	2.191	3.810	20.000	55.385	140.000	25.295	18.574	14.704	23.463
UFR_BW (ml/h-kg)										
T1A	435	0.037	0.129	0.427	1.493	9.000	0.585	0.429	0.399	0.460
T1B	467	0.006	0.116	0.486	2.034	6.000	0.675	0.472	0.437	0.510
T2	50	0.032	0.063	0.276	0.994	2.017	0.389	0.276	0.215	0.353
T3	53	0.012	0.076	0.279	1.199	2.143	0.375	0.267	0.211	0.339
ER_CRE (mg/h)										
T1A	474	1.725	6.338	23.259	66.705	634.480	30.327	22.277	20.799	23.860
T1B	488	1.489	5.546	21.854	61.595	499.548	28.717	21.441	20.009	22.977
T2	53	3.895	4.140	17.854	59.206	65.882	23.584	18.206	14.787	22.416
T3	57	0.346	5.062	22.352	90.980	119.796	28.770	20.210	15.665	26.075
T5	37	2.118	2.698	21.199	77.434	118.778	26.893	18.465	13.484	25.285
ER_CRE_BW (mg/h-kg)										
T1A	434	0.025	0.094	0.337	0.901	7.931	0.443	0.326	0.303	0.350
T1B	459	0.024	0.074	0.331	0.904	6.244	0.425	0.319	0.297	0.343
T2	49	0.049	0.063	0.264	0.759	1.209	0.330	0.253	0.203	0.315
T3	53	0.003	0.063	0.244	1.022	1.630	0.363	0.253	0.192	0.332
SG										
T1A	512	1.0013	1.0052	1.0166	1.0285	1.0327	1.0165	1.0165	1.0159	1.0171
T1B	544	1.0014	1.0049	1.0159	1.0277	1.0322	1.0158	1.0158	1.0151	1.0164
T2	70	1.0033	1.0050	1.0162	1.0272	1.0287	1.0162	1.0162	1.0146	1.0177
T3	71	1.0037	1.0078	1.0168	1.0259	1.0278	1.0171	1.0171	1.0157	1.0184
T5	63	1.0040	1.0044	1.0203	1.0287	1.0326	1.0191	1.0191	1.0172	1.0210
CRE (mg/dl)										
T1A	510	8.145	22.398	84.898	193.778	322.624	92.514	75.406	71.017	80.067
T1B	534	4.525	18.778	80.034	182.353	312.104	86.264	69.274	65.139	73.672
T2	69	15.611	25.792	91.629	196.946	250.679	100.644	84.131	72.039	98.252
T3	71	10.294	27.941	109.842	217.195	265.045	112.515	96.558	83.454	111.720
T5	48	16.177	23.529	107.296	214.593	301.131	114.187	94.341	77.343	115.074

*T1A* weekday under 20 weeks pregnant, *T1B* weekend day under 20 weeks pregnant, *T2* 2nd trimester, *T3* 3rd trimester, *T5* 2–3 months postpartum

**Fig. 2:**
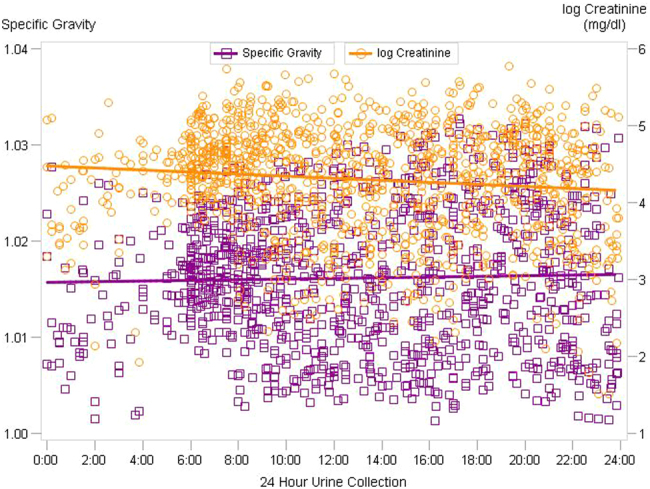
**Scatterplot of creatinine and specific gravity by time of day**. Scatterplot of specific gravity and natural log transformed creatinine (mg/dl) by time of day for all maternal urine samples collected

**Fig. 3:**
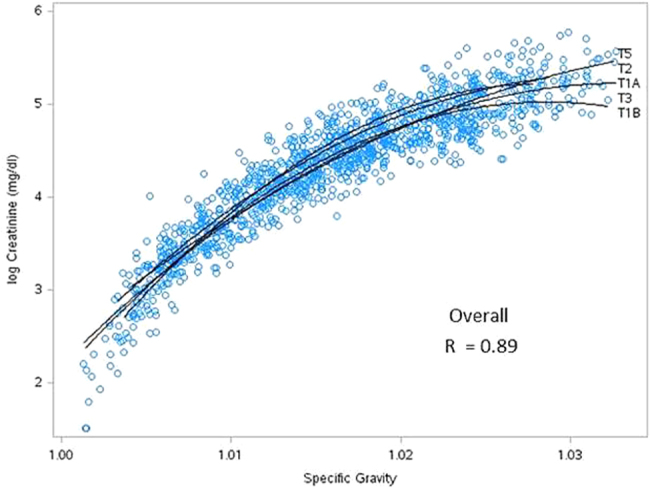
**Creatinine vs specific gravity by pregnancy time point**. Scatterplot between natural log transformed creatinine (mg/dl) and specific gravity for all maternal urine samples collected. Regression lines show the curvilinear relationship for each sampling period (T1A: <20 weeks gestation week day; T1B: <20 weeks gestation weekend day; T2: 24–28 weeks gestation; T3: 32–36 weeks gestation; T5: 2–3 months post-partum)

To visualize how specific gravity and creatinine vary across time, Fig. 2 shows a scatterplot of natural log transformed creatinine and specific gravity by time of day for all maternal urine samples collected. The regression lines show how specific gravity remains more constant across a 24 h day than creatinine, which seems to decrease in concentration throughout the day. When collection time was considered continuously, the mixed model results, with BMI included as a covariate, indicated a significant decrease (*p* value = 0.0023) in creatinine concentration and no change in specific gravity (*p* value = 0.1286). There was a clear curvilinear relationship between log creatinine and specific gravity for all pregnancy periods, as shown in Fig. 3. The equation for the curved line that best fits the data points $\left( {\mathrm{{log}}\,\mathrm {CRE} = - 3336 + 6483\,\mathrm {SG} - 3144\,\mathrm {SG}^2} \right)$ produced unbiased and homoscedastic residuals with a coefficient of determination (*R*^2^) equal to 0.89. This curve shows that log creatinine concentrations do not increase linearly with increasing specific gravity.

The average age of participants was 32.4 years, with 89% having a college or university degree, 46% in their first pregnancy and about 32% having never smoked (Table 2). The association between each maternal characteristic and urinary measure was examined using linear mixed models. None of the variables were found to be significant predictors of the urine dilution metrics, except for BMI. In our study, close to 72% of the women were considered of normal weight or underweight, having a prepregnancy BMI below 25, while 28% were considered overweight or obese, having a BMI of 25 or higher. The results in Table 3 show that urine flow rate decreased as BMI increases, while specific gravity, creatinine and creatinine excretion rate showed an increase with increasing BMI. Overweight or obese women had 0.22% higher specific gravity levels but 23% higher creatinine concentrations.

**Table 2:** Characteristics of participants in the P4 study

	# Participants	% Of Participants	# Observations
Born in Canada			
Yes	63	78.8	1023
No	17	21.3	237
Maternal age (years)			
<30	17	21.5	218
30–35	36	45.6	588
>35	26	32.9	436
BMI			
Underweight/normal (BMI < 25)	53	71.6	590
Overweight/obese (BMI ≥ 25)	21	28.4	470
Parity			
0	37	46.3	546
1	34	42.5	569
2+	9	11.3	145
Household income			
<100K	31	41.3	468
100K+	44	58.7	732
Maternal education			
<University/college	9	11.3	114
College diploma	14	17.5	198
University degree	36	45.0	590
Masters or PhD	21	26.3	358
Occupation			
Health care workers	15	18.8	254
Office workers (inc. Gov)	30	37.5	472
Unemployed	14	17.5	198
All others	21	26.3	336
Smoking status			
Never smoked	53	68.0	825
Ever smoked	25	32.1	402

**Table 3:** Least squares GM, 95% CIs, % changes and *p* values by BMI category from mixed models for urinary flow rate, creatinine excretion rate, SG and creatinine in all maternal urine samples.

	LS GM (95% CI)	% Change	*p* value
Urinary flow rate (ml/h)			
Underweight/normal (BMI < 25)	30.39 (26.56, 34.77)	–	–
Overweight/obese (BMI ≥ 25)	25.54 (22.12, 29.49)	−18.99	0.0562
Creatinine excretion rate (mg/h)			
Underweight/normal (BMI < 25)	20.29 (17.92, 22.97)	–	–
Overweight/obese (BMI ≥ 25)	23 (20.13, 26.29)	11.80	0.1398
Specific gravity			
Underweight/normal (BMI < 25)	1.0154 (1.0142, 1.0166)	–	–
Overweight/obese (BMI ≥ 25)	1.0176 (1.0164, 1.0188)	0.22	**0.0042**
Creatinine (mg/dl)			
Underweight/normal (BMI < 25)	67.45 (60.25, 75.52)	–	–
Overweight/obese (BMI ≥ 25)	87.77 (77.83, 98.97)	23.15	**0.0006**

Significant *p* values (<0.05) are shown in bold

To evaluate how these measures changed throughout a day and across pregnancy, Table 4 shows the geometric means, percent changes and *p* values generated from mixed models with BMI included in the model as a covariate. The *p* values refer to differences with respect to the reference category, as indicated in the table. No interaction terms were retained in the model, as they were all insignificant and did not improve model fit. The results showed that urinary flow and creatinine excretion rates increased throughout the day and were significantly higher between 4:00 p.m. and midnight. These rates were also higher in the first trimester than the second and third trimesters; however, these differences in creatinine excretion rates were only significant when BMI was included in the model. Creatinine concentration decreased throughout the day with significantly higher concentrations between midnight and 8:00 a.m. and significantly lower concentrations were measured during the early pregnancy period. Specific gravity, on the other hand shows no systematic trend within a day or across pregnancy.

**Table 4:** Time trends for urinary flow rate, creatinine excretion rate, specific gravity and creatinine in all maternal urine samples

	UFR (ml/h)			ER_CRE (mg/h)		
	LS GM (95% CI)	% Change	*p* value	LS GM (95% CI)	% Change	*p* value
Collection time						
00:00–7:59	18.29 (16.03, 20.87)	−34.00	**<0.0001**	15.31 (13.48, 17.4)	−30.61	**<0.0001**
8:00–15:59	27.71 (24.72, 31.07)	–	–	22.07 (19.77, 24.63)	–	–
16:00–23:59	36.23 (32.19, 40.78)	30.74	**<0.0001**	25.94 (23.16, 29.05)	17.54	**0.0011**
Pregnancy period						
T1A	29.08 (25.77, 32.8)	43.64	**0.0018**	22.66 (20.25, 25.35)	28.09	**0.0275**
T1B	30.13 (26.75, 33.93)	48.85	**0.0006**	22.1 (19.78, 24.69)	24.91	**0.0482**
T2	17.94 (14.31, 22.5)	−11.35	0.4135	17.11 (13.74, 21.32)	−3.25	0.8187
T3	20.24 (16.11, 25.43)	−	–	17.69 (14.21, 22.02)	–	–
Collection day						
Weekend	29.24 (25.98, 32.91)	–	–	21.63 (19.36, 24.16)	–	–
Weekday	26.72 (23.81, 29.99)	−8.62	0.0729	21.58 (19.38, 24.03)	−0.21	0.9647

	SG			CRE (mg/dl)		
	LS GM (95% CI)	% Change	*p* value	LS GM (95% CI)	% Change	*p* value
Collection time						
00:00–7:59	1.0162 (1.0151, 1.0174)	−0.02	0.7608	83.76 (74.68, 93.94)	7.58	0.1577
8:00–15:59	1.0164 (1.0154, 1.0174)	−	–	77.86 (70.48, 86.01)	−	−
16:00–23:59	1.0168 (1.0158, 1.0179)	0.05	0.282	71.54 (64.49, 79.35)	−8.12	0.0565
Pregnancy period						
T1A	1.0167 (1.0157, 1.0178)	0.04	0.6353	77.07 (69.62, 85.31)	−13.79	0.0925
T1B	1.0163 (1.0153, 1.0173)	0.00	0.9972	73.13 (66.15, 80.84)	−18.20	**0.0225**
T2	1.0165 (1.0148, 1.0182)	0.02	0.8505	85.68 (72.09, 101.83)	−4.16	0.7012
T3	1.0163 (1.0146, 1.0181)	–	–	89.4 (75.02, 106.53)	–	–
Collection day						
Weekend	1.0163 (1.0153, 1.0173)	–	–	73.91 (66.94, 81.62)	–	–
Weekday	1.0166 (1.0157, 1.0176)	0.03	0.3958	79.56 (72.26, 87.6)	7.64	0.0693

Least squares GMs, 95% CIs, % changes and *p* values were generated from mixed models adjusting for BMI. Significant *p* values (<0.05) are shown in bold

The results for the ICCs, shown in Table 5, indicate what proportion of the total variation in each of the dilution measures is accounted for by the variation among individuals. In general, the ICCs were quite similar for the weekdays, the weekend days and across all study periods. Bodyweight-adjusted urinary flow rate had slightly higher ICCs than urinary flow rate, while bodyweight-adjusted creatinine excretion rate had lower ICCs than creatinine excretion rate. In terms of reproducibility, the results in all measures showed low reproducibility, both within a day and throughout the entire study period. Specific gravity results were slightly larger than the other measures, with the largest being for a weekend day, having an ICC of 0.38.

**Table 5:** Intraclass correlation coefficients (ICCs) and 95% confidence intervals

	# Participants	# Observations	ICC (95% CI)
UFR (ml/h)			
T1a (weekday)	63	475	0.23 (0.15, 0.34)
T1b (weekend)	65	498	0.25 (0.16, 0.36)
Across all time points	80	1132	0.23 (0.17, 0.32)
UFR_BW (ml/h-kg)			
T1a (weekday)	57	435	0.26 (0.17, 0.38)
T1b (weekend)	60	467	0.27 (0.18, 0.39)
Across all time points	80	1005	0.26 (0.19, 0.35)
ER_CRE (mg/h)			
T1a (weekday)	63	474	0.24 (0.15, 0.35)
T1b (weekend)	65	488	0.29 (0.20, 0.40)
Across all time points	80	1109	0.23 (0.17, 0.31)
ER_CRE_BW (mg/h-kg)			
T1a (weekday)	57	434	0.22 (0.13, 0.34)
T1b (weekend)	60	459	0.26 (0.17, 0.38)
Across all time points	80	995	0.23 (0.16, 0.31)
SG			
T1a (weekday)	63	510	0.32 (0.23, 0.43)
T1b (weekend)	67	534	0.38 (0.29, 0.49)
Across all time points	80	1260	0.28 (0.21, 0.37)
CRE (mg/dl)			
T1a (weekday)	63	510	0.26 (0.18, 0.37)
T1b (weekend)	67	534	0.30 (0.21, 0.41)
Across all time points	80	1232	0.25 (0.19, 0.33)

**Fig. 4:**
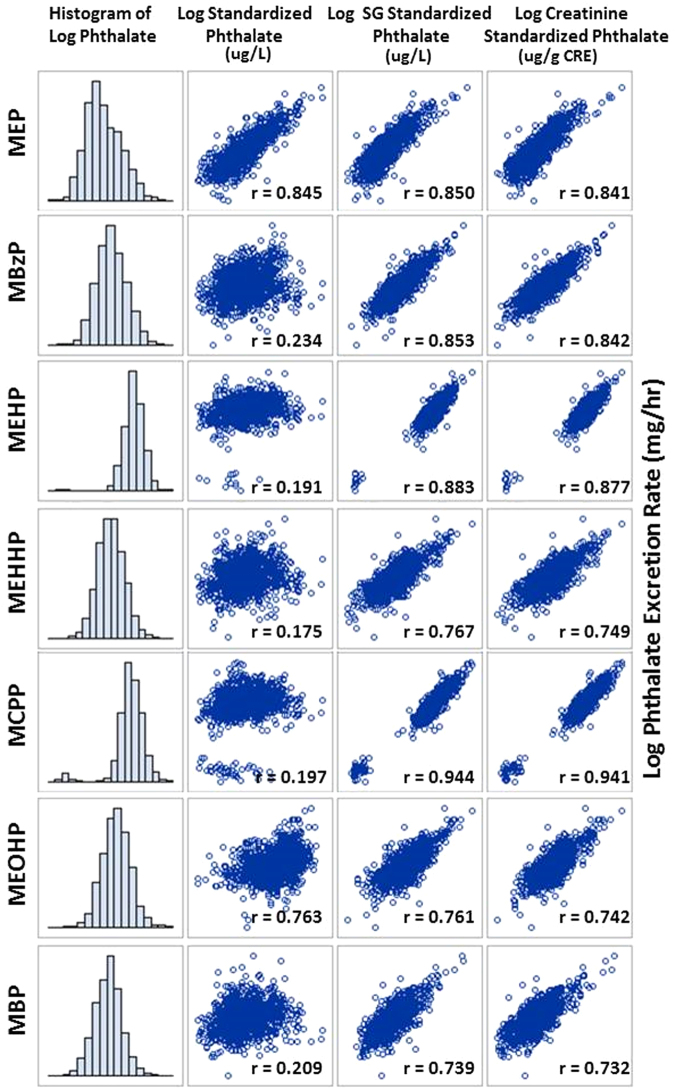
**Correlation between standardized phthalate concentrations and the excretion rate**. Histograms illustrating the distribution of the log transformed phthalate metabolites and scatterplots depicting the correlation between each of the creatinine (µg/g CRE) and specific gravity (µg/L) standardized phthalate concentrations and the log phthalate excretion rate (mg/hr)

The excretion rate of each of the phthalates was calculated and the measured concentrations were normalized using the specific gravity and creatinine procedures. All MEP, MBP and MEHHP concentrations exceeded the limit of detection. For MEHP, MBzP, MCPP and MEOHP, at least 93% of the concentrations were above the limit of detection. The machine readings from the lab were used and concentrations of 0 were substituted with 0.0001 in order to log transform the skewed phthalate distributions. The histograms and scatterplots of the log transformed values are shown in Fig. 4, illustrating the distribution of the phthalates and the correlation between each of the creatinine and specific gravity-standardized phthalate concentrations and the excretion rate. Both the SG and the creatinine-standardized phthalates were all highly correlated with the excretion rate, ranging from *r* = 0.73 for creatinine-standardized MBP to *r* = 0.94 for SG-standardized MCPP. Although correlation coefficients for both techniques are very similar, the SG-standardized concentrations are slightly higher for all seven phthalates.

## Discussion

In epidemiologic studies, biomarkers of exposure are usually measured in single spot urine samples. To minimize the effect of variations in the dilution of the samples, the concentration of a chemical is routinely normalized according to reference parameters, such as specific gravity or creatinine. The required features of the parameter are that it should be subject to only minimal inter-individual variations, should have a constant day-to-day excretion and be little influenced by exogenous factors such as quantity of urine, diet, physical activity, etc [17]. The objective of this study was to examine the physiological and temporal characteristics affecting urinary dilution in pregnant women and to determine the best practice for correcting for the dilution of urinary biomarker concentrations.

This study demonstrated that urinary flow rates vary greatly by time of day, with the highest rates occurring during the daytime. Dilution of urine will affect the urinary concentration of the biomarkers leading to incorrect conclusions on the extent of exposure. A study of healthy, non-smoking adult men and women also found that urinary flow rates are highest during the day [18]. Subsequently, they advised that if the excretion rate of a chemical is lower in first morning voids than in 24 h urine samples, it may be due to increased excretion of the chemical sampled during the day because of increased urinary flow rate. As a result, first morning voids may underestimate the true chemical excretion [18]. Creatinine concentrations were significantly higher overnight than in the daytime when flow rate was higher. These results support the findings by Trachtenberg et al. [19], who also found that in urine samples of children, creatinine concentration varies inversely with urinary flow rate. By adjusting for creatinine in biomarker concentrations, one is assuming that the excretion rate of creatinine is constant, which was not the case in their study or in ours. A biomarker’s excretion would need to increase with the flow rate at the same rate as creatinine in order to not introduce a flow rate bias [19].

Specific gravity and creatinine are two of the primary methods used for adjusting urinary concentration for dilution and for this reason many studies have investigated the relationship between the two measures. One study of 534 men and women reported a high correlation of 0.82 between creatinine and specific gravity in spot urines, with no significant intra- or inter-day variations for these two parameters [20]. Another analysis of over 10,000 paired urine samples reported a correlation of 0.84 [21]. Pearson's correlation coefficient of 0.83 was reported between the log transformed creatinine and specific gravity results from a large study that analyzed 20,395 urinary samples collected between 1985 and 2010 [22]. While many of these studies report the linear correlation coefficient, the curvilinear relationship was evident in many of the graphical representations. Our coefficient of determination, *R*^2^ = 0.89, implies that 89% of the variation in creatinine is explained by the curvilinear association between specific gravity and creatinine.

The assumption underlying the creatinine correction approach is that creatinine excretion in urine occurs at a rate that is less variable than the rate of urinary flow in volume excreted per time [4]. However, age, sex, race, BMI and to a lesser extent time of day when the urine was collected have been identified as significant predictors of urinary creatinine concentration [23]. A diurnal pattern with overnight and early morning urine samples containing less creatinine than late afternoon samples has also been described [24]. Specific gravity-standardized concentrations appear to be less dependent on body size, age and sex than creatinine [25]. The women in our study were homogeneous with respect to race and age, with most being Canadian born with a mean age of 32.4 years. The only significant systematic variation was observed across categories of BMI, suggesting that when adjusting urinary contaminant concentrations for hydration status in pregnant women, BMI should also be considered. There was a significant time of day effect for all measures except specific gravity, which is not surprising given that normal urine specific gravity usually ranges from 1.013 to 1.029 [26] and are not expected to have large fluctuations. A trend was also observed throughout pregnancy for all except specific gravity, indicating that within this pregnant cohort, testing for time differences in contaminant levels among individuals, specific gravity is less influenced by temporal variables and offers a dependable method of adjusting for urinary dilution.

We found low reproducibility for all urinary dilution measures throughout pregnancy and across a day, with specific gravity showing slightly higher reproducibility. The low ICCs indicate that in order to accurately represent urine flow rates, creatinine excretion rates and specific gravity over the course of a day or throughout pregnancy, a single spot urine sample may not suffice and more than one measurement at different times of day or at different stages throughout pregnancy may be required to get a more accurate picture of urinary dilution. Another study used data from the US National Health and Nutrition Examination Survey (NHANES) to examine the suitability of using creatinine concentrations or specific gravity for urinary correction in exposure assessment. They also found that interpersonal specific gravity variability is less than creatinine and may be a more appropriate method of correction [27]. Comparisons of creatinine-standardized concentrations of biomarkers in populations can be affected by variability in creatinine excretion rates; similarly, variability in urinary flow rates may affect volumetric concentrations [28]. Fortin et al. [28] reported that the between void variability in creatinine excretion rate and urinary flow rate during the course of a day could have a profound impact on interpreting the estimated absorbed dose. If the health outcome under study is also independently associated with any characteristics such as age, BMI and race, then confounding or systematic bias can occur if creatinine-standardized urinary concentrations are used [4].

For chemicals with short half-lives that are primarily excreted in urine, it is assumed that the urinary excretion is directly proportional to the rate of intake of the chemical on average [4]. In comparing the adjustment techniques of the phthalate concentrations during pregnancy, we were able to show that the specific gravity-standardized concentrations were slightly more highly correlated with the calculated excretion rate of each of the phthalates. Better correlations between true urinary excretion rates have been reported for specific gravity-standardized urinary concentrations compared to creatinine standardized [5]. Several studies agree that in order to correct for urine dilution of phthalate metabolites, specific gravity rather than creatinine may be more appropriate, especially for populations undergoing physiological changes in renal function such as pregnant women [14, 29, 30]. Furthermore, in pregnancy, creatinine clearance is greatly increased, as is urine volume [30].

One of the challenges of using urinary biomarkers is the potential for kidney function to affect the biomarker levels in the body. The concentration of a chemical in the blood or urine may be impacted by the GFR, the ability of the kidney to filter the chemicals and the ability of the kidney tubules to secrete or reabsorb the chemicals [2]. This is particularly important in human pregnancy, where effective renal plasma flow and glomerular filtration rate increase to levels 50–80% above non-pregnant values. The increments occur shortly after conception, persist throughout the second trimester and are slightly reduced in late pregnancy [31]. This presents a disadvantage of using creatinine adjustment methods because it undergoes substantial processing in the kidney, leading to the potential for kidney tubule processing to impact creatinine concentrations in urine. Furthermore, creatinine is affected by muscle mass and diet, thus resulting in variations that are unrelated to hydration status [2]. We have shown that urine specific gravity standardization has lower intra-individual variability compared to creatinine. The same has also been found when compared to urine osmolality measurements [26]. Specific gravity-standardized concentrations also appear to be less dependent on body size, age and sex than creatinine [25]. In our study, specific gravity systematically varied with BMI, which is important to consider in studies involving BMI or BMI-related outcomes. The systematic variations in the specific gravity-standardized concentrations, as a function of BMI, can introduce a confounding bias when tested against these outcomes. Multivariable models that include specific gravity, as well as BMI, as covariates, will be able to account for both hydration status and BMI, as well as be able to assess their interaction. Another notable disadvantage of urine specific gravity is that it is affected by the number and size of particles in the solution such that a highly concentrated urine would be falsely concluded when unusual quantities of larger molecules such as glucose, proteins or urea are present [26]. During pregnancy, the reabsorption of glucose in the proximal and collecting tubules is less effective, with variable excretion. Due to the increases in GFR, the excretion of protein may increase, but in normal pregnancies the total urinary protein concentration does not increase above the upper normal limit [32].

The main strength of our study was the sampling schedule and the extensive information collected in numerous questionnaires and diaries. Collecting multiple urine samples at different times of the day and at several stages throughout pregnancy allowed us to test for diurnal and across pregnancy differences, and investigate any systematic trends across demographic groups of interest, such as age or BMI. The repeated measures from each participant provided us with information on between- and within-subject variability across a day and throughout pregnancy. The data collected on urine volume and time since last void allowed us to directly calculate the urinary flow rate and, subsequently, the excretion rate of several phthalates and compare them with the various hydration-adjusted concentrations.

Our study did not show any significant systematic variations of urinary flow rate, observed across demographic categories, but it did vary with time. If there had been systematic differences between categories, as was previously shown by Hays et al. [4], then urinary concentrations would be impacted by systematic differences in urinary flow rate, in addition to differences in exposure levels. These differences in concentrations cannot be assumed to be random with respect to health outcomes or populations of interest [4]. If the health outcome is also independently associated with any of these characteristics, then confounding or systematic bias can occur if the analyte excretion rate (calculated using urinary flow rate) is used as a measure of exposure. Barr et al. [23] have recommended that the urinary dilution measure be entered as an independent variable in regression analyses rather than applied as a hydration status “correction” factor to measured urinary analyte concentrations.

In biomonitoring studies, the urinary flow rate corresponds to the volume of urine accumulated in the bladder over the time since last void. The validity of timed urine samples is much dependent on complete voiding of the bladder, fully collecting the urine void and accurately recording the volume and time of collection and precise recording of the timing between each void. A major limitation of this study is the significant potential for incomplete urine void collections. The urine volumes ranged from 10 to 120 ml per void, suggesting that there may be incomplete voiding of the bladder or incomplete collection of the voided volume; both of which may contribute to some of the intra-day variability in urinary flow rates [24] and lead to unreliable excretion rate calculations. Furthermore, the potential bias introduced by the measurement error in urinary flow rate and creatinine excretion rate can increase the standard errors and this loss of precision could lead to a decrease in the power to detect associations with the demographic variables Furthermore, the generalizability of this homogenous cohort is limited, as it is biased towards highly educated, high-income, Caucasian women.

The outcome of this analysis suggests that specific gravity adjustment is a favorable approach for correcting for the hydration status of the pregnant women from this cohort. While most researchers agree that adjustment techniques are necessary, given the disadvantages and limitations discussed, some authors have advocated not adjusting for hydration status at all [33, 34]. It has also been recommended that unadjusted analyte concentrations be modeled as the dependent variable and the urinary creatinine concentration be included in the multiple regression as an independent variable, allowing other variables in the model to be independent of the effects of creatinine [23]. A similar approach has been used for specific gravity [35, 36]. Treating creatinine as a covariate (if including it at all) may lead to less bias in modeling results of exposure–outcome associations [37]. Although more research is required to verify its validity, others have recommended a modified specific-gravity-adjusted-creatinine ratio-normalization technique or the creatinine-regression normalization technique to obtain the best agreement between spot and simulated 24 h urine results [3]. To restate what Heavner et al. [3] have concluded, renal excretion mechanisms are chemical specific and investigators require a thorough understanding of the relationship between the biomarker concentration and excretion rate, urine flow, specific gravity and creatinine concentration to avoid using normalization techniques that may be inappropriate.
